# Time to clearance of *Chlamydia trachomatis* RNA and DNA after treatment in patients coinfected with *Neisseria gonorrhoeae* – a prospective cohort study

**DOI:** 10.1186/s12879-016-1878-3

**Published:** 2016-10-11

**Authors:** Carolien M. Wind, Maarten F. Schim van der Loeff, Magnus Unemo, Rob Schuurman, Alje P. van Dam, Henry J. C. de Vries

**Affiliations:** 1 STI Outpatient Clinic, Department of Infectious Diseases, Public Health Service Amsterdam, PO Box 2200, 1000 CE Amsterdam, The Netherlands; 2 Department of Dermatology, Academic Medical Center, University of Amsterdam, Amsterdam, The Netherlands; 3 Department of Infectious Diseases, Public Health Service Amsterdam, PO Box 2200, 1000 CE Amsterdam, The Netherlands; 4 Center for Infection and Immunity Amsterdam, Academic Medical Center, University of Amsterdam, Amsterdam, The Netherlands; 5 WHO Collaborating Centre for Gonorrhoea and Other STIs, National Reference Laboratory for Pathogenic Neisseria, Department of Laboratory Medicine, Microbiology, Örebro University, SE-701 85 Örebro, Sweden; 6 Department of Medical Microbiology, University Medical Centre Utrecht, PO Box 85500, 3508 GA Utrecht, The Netherlands; 7 Public Health Laboratory, Public Health Service Amsterdam, Amsterdam, The Netherlands; 8 Department of Medical Microbiology, Onze Lieve Vrouwe Gasthuis General Hospital, Amsterdam, The Netherlands

**Keywords:** *Chlamydia trachomatis*, *Neisseria gonorrhoeae*, Antimicrobial resistance, Nucleic acid amplification test, Test of cure

## Abstract

**Background:**

Performing a test of cure (TOC) could demonstrate success or failure of antimicrobial treatment of *Chlamydia trachomatis* infection, but recommendations for the timing of a TOC using nucleic acid amplification tests (NAATs) are inconsistent. We assessed time to clearance of *C. trachomatis* after treatment, using modern RNA- and DNA-based NAATs.

**Methods:**

We analysed data from patients with a *C. trachomatis* and *Neisseria gonorrhoeae* coinfection who visited the STI Clinic Amsterdam, The Netherlands, from March through October 2014. After treatment with ceftriaxone plus either azithromycin or doxycycline, patients self-collected anal, vaginal or urine samples during 28 consecutive days. Samples were analysed using an RNA-based NAAT (Aptima Combo 2) and a DNA-based NAAT (Cobas 4800 CT/NG). We defined clearance as three consecutive negative results, and defined “blips” as isolated positive results following clearance.

**Results:**

We included 23 patients with *C. trachomatis* and *N. gonorrhoeae* coinfection. All patients cleared *C. trachomatis* during follow-up, and we observed no reinfections. The median time to clearance (range) was 7 days (1–13) for RNA, and 6 days (1–15) for DNA. Ninety-five per cent of patients cleared RNA at day 13, and DNA at day 14. The risk of a blip after clearance was 4.4 % (RNA) and 1.7 % (DNA).

**Conclusions:**

If a TOC for anogenital chlamydia is indicated, we recommend performing it at least 14 days after initiation of treatment, when using modern RNA- and DNA-based assays. A positive result shortly after 14 days probably indicates a blip, rather than a treatment failure or a reinfection.

## Background

*Chlamydia trachomatis* is the most common bacterial sexually transmitted infection (STI) globally, leading to late sequelae like pelvic inflammatory disease and infertility [1, 2]. Currently, the first-choice treatment for anogenital chlamydia consists of a single 1000 mg dose of azithromycin, or 100 mg doxycycline twice daily for 7 days [3, 4]. No resistance of *C. trachomatis* to either of these drugs has been reported, and a recent randomized controlled trial suggested no inferiority of azithromycin (97 % effective) compared to doxycycline (100 % effective) in urogenital chlamydia infections [5]. However, some studies voice concern about the efficacy of azithromycin as first-choice treatment for anorectal chlamydia [6–9]. Persisting *C. trachomatis* infections could be detected by performing a test of cure (TOC) after treatment. Current chlamydia treatment guidelines recommend a TOC between 3 and 4 weeks after initiation of treatment, in certain patient groups or when symptoms persist [4, 7, 10]. However, up to 90 % of chlamydia infections are asymptomatic, which could lead to persisting infections remaining undetected [4, 11, 12]. Previous reports on the appropriate timing of a TOC using molecular methods are inconsistent, and show (intermittent) persistence of *C. trachomatis* nucleic acids between 0 and 42 % up to 51 days after treatment [9, 13–19]. Recently, we performed a prospective cohort study on time to clearance for *N. gonorrhoeae*, using modern RNA- and DNA-based nucleic acid amplification tests (NAATs) [20]. Thirty-seven per cent of the included patients were also coinfected with *C. trachomatis*. As this study has results of 28 consecutive days for both RNA and DNA, we evaluated the appropriate timing of TOC for anogenital *C. trachomatis* infections in these coinfected patients.

## Methods

### Study population and procedure

In a previously performed cohort study we included patients with anogenital *N. gonorrhoeae* infection, who visited the STI Outpatient Clinic in Amsterdam, The Netherlands, from March through October 2014 [20]. We collected follow-up data and samples for only one infected anatomical site. The Academic Medical Center Amsterdam medical ethics committee approved the original cohort study (NL45935.018.13), and all patients provided written informed consent. For the current analysis, only patients coinfected with *C. trachomatis* were included from the cohort.

All patients had received routine treatment for *N. gonorrhoeae* and *C. trachomatis* coinfection, consisting of a single intramuscular dose of 500 mg ceftriaxone, plus one oral dose of azithromycin 1000 mg in case of urogenital infection, or doxycycline 100 mg twice daily for at least 7 days in case of anorectal infection. Participants self-collected urine, anal or vaginal NAAT samples: one for RNA-based and one for DNA-based NAAT. Samples were collected pre-treatment and subsequently daily for 28 consecutive days after treatment. We requested participants to abstain from sexual contact or use condoms, refrain from vaginal or rectal douching and keep a study diary. At the end-of-study visit (within 35 days of inclusion) a nurse collected samples from the designated anatomical site for both NAATs.

### NAAT testing for *C. trachomatis*

Samples for the RNA-based NAAT were tested using the Aptima Combo 2 assay for *C. trachomatis* and *N. gonorrhoeae* (Hologic, San Diego, California). Test sensitivity is 93–98 % and specificity is >99 % [21–23]. Equivocal results were retested using the Aptima CT assay (Hologic). We considered repeatedly equivocal results as positive and excluded samples with repeatedly invalid results.

Samples for DNA-based NAAT were tested using the Cobas 4800 CT/NG assay for *C. trachomatis* and *N. gonorrhoeae* (Roche, Basel, Switzerland), and reported as either negative or positive with corresponding cycle threshold (Ct) value. Test sensitivity is 87–97 % and specificity is >99 % [22–24]. Pre-treatment samples with discrepant RNA and DNA results were retested, using the Aptima CT assay (Hologic) for RNA samples, and the Abbott RealTime CT/NG assay (Abbott, Abbott Park, Illinois) for DNA samples.

### Statistical analysis

The primary endpoint, clearance of *C. trachomatis* using RNA- or DNA-based NAAT, was defined as three or more consecutive negative results following a positive result. We allowed one missing sample between the last positive and the first negative result. Reinfection was defined as positive test results on three or more consecutive days after clearance; tests had to be positive for both RNA and DNA on at least 1 day. To analyse differences we compared patients grouped by anatomical site using Chi-square, Fisher’s exact or Kruskal-Wallis testing. Time to clearance was analysed with Kaplan-Meier curves, log-rank testing and Cox regression analysis. If we could not determine the exact day of clearance due to missing samples, the patient was excluded from this analysis.

The secondary endpoint, intermittent presence of RNA or DNA (“blip”), was defined as a positive test following clearance, not due to reinfection. Only positive results after the three consecutive negative tests results, used to define clearance, were eligible as a blip. We used logistic regression with generalized estimated equation (GEE) models to identify characteristics associated with blips. All analyses were performed using Stata (version 13; StataCorp, College Station, Texas).

## Results

### Participants

Out of 462 patients with anogenital gonorrhoea diagnosed at our STI clinic from March through October 2014, 77 were included in the original cohort. Twenty-six patients (34 %) had a coinfection with *C. trachomatis* of whom three were lost to follow-up. The remaining 23 patients were included in the current analysis.

### Baseline characteristics

We included nine women, all with endocervical infections, and 14 men, of whom seven had a urethral and seven a rectal infection; 71 % were men who have sex with men (MSM, Table 1). The median age was 24 years (interquartile range [IQR]: 20–35 years); women were significantly younger (median 20 years) compared to men (median 32 years, *P* < 0.001). Five men (22 %) were HIV-positive, and four (80 %) were on antiretroviral therapy, of whom three had CD_4_^+^ cell counts of ≥500 cells/mm^3^. A previous chlamydia infection was reported by 12 patients (52 %), and 13 (57 %) currently experienced symptoms. The median time between diagnosis and inclusion was 8 days (range 0–12). At inclusion 16 patients (70 %) received ceftriaxone with azithromycin, and seven (30 %) received ceftriaxone with doxycycline.

**Table 1:** Baseline characteristics of 23 patients with *Chlamydia trachomatis* and *Neisseria gonorrhoeae* coinfection at inclusion

Characteristics	Total N (%)^a^	Urethra N (%)^a^	Rectum N (%)^a^	Endocervix N (%)^a^	*P*
Total	23	7 (30.4)	7 (30.4)	9 (39.1)	
Gender					
Male	14 (60.9)	7 (100.0)	7 (100.0)	0 (0.0)	
Female	9 (39.1)	0 (0.0)	0 (0.0)	9 (100.0)	
Median age, in years (IQR)	24 (20–35)	29 (24–35)	40 (24–44)	20 (19–23)	0.003
Ethnicity					1.00
Dutch	11 (47.8)	3 (42.9)	4 (57.1)	4 (44.4)	
Non-Dutch	12 (52.2)	4 (57.1)	3 (42.9)	5 (55.6)	
Sexual risk group					
MSM	10 (43.5)	3 (42.9)	7 (100.0)	0 (0.0)	
Hetero male	4 (17.4)	4 (57.1)	0 (0.0)	0 (0.0)	
Female	9 (39.1)	0 (0.0)	0 (0.0)	9 (100.0)	
HIV positive	5 (21.7)	1 (14.3)	4 (57.1)	0 (0.0)	0.02
Using cART	4 (80.0)	0 (0.0)	4 (100.0)	-	0.20
CD_4_^+^ cell count (cells/mm^3^)					1.00
350–499	1 (20.0)	0 (0.0)	1 (25.0)	-	
≥ 500	4 (80.0)	1 (100.0)	3 (75.0)	-	
Previous chlamydia episode	12 (52.2)	3 (42.9)	4 (57.1)	5 (55.6)	1.00
Chlamydia in preceding 6 months	3 (13.0)	0 (0.0)	1 (14.3)	2 (22.2)	0.75
Symptoms or signs at examination^b,c^	13 (56.5)	6 (85.7)	3 (42.9)	4 (44.4)	0.23
Median time to inclusion, days (IQR)	8 (0–12)	0 (0–0)	10 (7–13)	9 (8–12)	0.003
Treatment at inclusion^d^					0.001
Ceftriaxone + azithromycin	16 (69.6)	7 (100.0)	1 (14.3)	8 (88.9)	
Ceftriaxone + doxycycline	7 (30.4)	0 (0.0)	6 (85.7)	1 (11.1)	

*IQR* interquartile range, *MSM* men who have sex with men, *HIV* human immunodeficiency virus, *cART* combination antiretroviral therapy

^a^Unless otherwise indicated

^b^Symptoms included: discharge, itch, burning, frequent or painful urination, bleeding, abdominal pain, pain during sex, anal cramps or pain, and changed defecation

^c^Signs included: red urethra, discharge, bleeding, fragile mucosa, swelling or anal ulcerations

^d^1 patient was negative for *Chlamydia trachomatis* at the initial visit and therefore received ceftriaxone mono-therapy. The test at inclusion was positive and doxycycline was started 6 days after inclusion; therefore the start of the study for the *C. trachomatis* analysis in this patient was day 6, and the treatment was ceftriaxone + doxycycline

### Behaviour after inclusion

The median number of collected samples was 28 (range 25–28, Table 2). Forty-eight per cent of patients missed at least one sample. Rectal or vaginal douching was reported by four of 16 patients with rectal or endocervical chlamydia (25 %). Sexual contact at some point during the 28 days of follow-up was reported by 12 patients (52 %), and condomless sex by five patients (22 %).

**Table 2:** Behaviour after inclusion and clearance of *Chlamydia trachomatis* based on RNA and DNA testing, by anatomical site

Characteristics	Total n (%)^a^	Urethra n (%)^a^	Rectum n (%)^a^	Endocervix n (%)^a^	*P*
Patients	23	7 (30.4)	7 (30.4)	9 (39.1)	
Behaviour after inclusion					
Median no. of samples collected (range)	28 (25–28)	28 (26–28)	28 (26–28)	27 (25–28)	0.01
Patients with missed samples	11 (47.8)	1 (14.3)	2 (28.6)	8 (88.9)	0.009
Median no. of missed samples (IQR)	1 (1–3)	2 (2–2)	1.5 (1–2)	1 (1–3)	0.86
Rectal/vaginal douching	4 (25.0)^g^	-	3 (42.9)	1 (11.1)	0.26
Sexual contact	12 (52.2)	3 (42.9)	4 (57.1)	5 (55.6)	1.00
Condomless sex	5 (21.7)	1 (14.3)	2 (28.6)	2 (22.2)	1.00
RNA clearance^b^					
Clearance during follow-up	23 (100.0)	7 (100.0)	7 (100.0)	9 (100.0)	
Day of clearance definable^c^	21 (91.3)	7 (100.0)	6 (85.7)	8 (88.9)	1.00
Median time to clearance, days (range)	7 (1–13)	5 (1–13)	6.5 (5–9)	8 (6–13)	0.20
Blips^d^					
Samples at risk for blip	411	140	126	145	
Number of blips	18	0	12	6	
Number of patients	8 (34.8)	0 (0.0)	3 (42.9)	5 (55.6)	0.09
Median time to first blip from being at risk, days (range)	1 (1–16)	-	1 (1–2)	4.5 (1–16)	0.61
DNA clearance^b,e^					
Clearance during follow-up	22 (100.0)	6 (100.0)	7 (100.0)	9 (100.0)	
Day of clearance definable^f^	21 (95.5)	6 (100.0)	7 (100.0)	8 (88.9)	
Median time to clearance, days (range)	6 (1–15)	6 (1–14)	5 (2–9)	7.5 (5–15)	0.08
Blips^f^					
Samples at risk for blip	403	117	138	144	
Number of blips	7	0	0	7	
Number of patients	5 (22.7)	0	0	5 (55.6)	0.01
Median time to first blip from being at risk, days (range)	3 (2–8)	-	-	3 (2–8)	
Mean Ct-value (range)	38.6 (35.3–41.7)	-	-	38.6 (35.3–41.7)	

*RNA* ribonucleic acid, *DNA* deoxyribonucleic acid; *IQR* inter-quartile range; *Ct* cycle threshold

^a^Unless otherwise indicated

^b^Based on a definition of 3 consecutive negative tests following a positive test

^c^For 2 patients the exact day of clearance could not be defined due to missing samples in the period of clearance

^d^Blip was defined as a positive test following clearance. Samples from all 23 patients were included; for those without an exact day of clearance due to missing samples, all samples after the first three consecutive negative results were considered at risk for blips

^e^One patient was excluded from this analysis because the sample at inclusion was negative for *Chlamydia trachomatis* DNA

^f^For 1 patient the exact day of clearance could not be defined due to missing samples in the period of clearance

^g^Rectal/vaginal douching was only reported on by the 16 patients with rectal/endocervical infection

### Clearance of *C. trachomatis* RNA and DNA

**Fig. 1:**
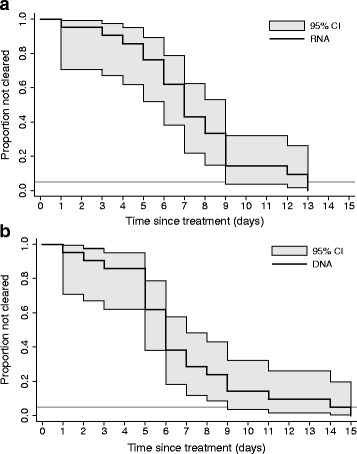
Time to clearance of *Chlamydia trachomatis* RNA (**a**) and DNA (**b**). The horizontal line represents 95 % clearance. *RNA*, ribonucleic acid; *DNA*, deoxyribonucleic acid

During the 28 days of follow-up all patients cleared *C. trachomatis* RNA, and none experienced a reinfection (Table 2). Because of missing samples in the days around clearance, we could not determine the exact day of clearance for two patients. The median time to clearance for the remaining 21 patients was 7 days (range 1–13), and 95 % of patients had cleared RNA at day 13 (Fig. 1a). One patient was post-hoc excluded from the DNA analysis because of a negative pre-treatment DNA result for *C. trachomatis*. All other patients cleared *C. trachomatis* DNA during follow-up, and there were no reinfections. We could not determine the exact day of clearance for one patient (Table 2). The median time to clearance of the 21 patients was 6 days (range 1–15), and 95 % of patients had cleared DNA at day 14 (Fig. 1b).

Because of the small sample size the power to detect associations with clearance is very limited. Cox regression analyses showed no significant associations with clearance, and Kaplan-Meier curves with log-rank testing showed no significant difference in clearance by anatomical site or treatment.

### Blips after clearance of *C. trachomatis*

After clearance of RNA, eight patients experienced 18 blips (Table 2). After clearance of DNA, five patients experienced seven blips, of which three (all vaginal samples) coincided with RNA blips (Table 2). Among the patients with blips, sex after clearance was reported by five (RNA) and three (DNA) patients. We observed both RNA and DNA blips among vaginal samples, while we observed only RNA blips among rectal samples, and no blips among urine samples.

**Fig. 2:**
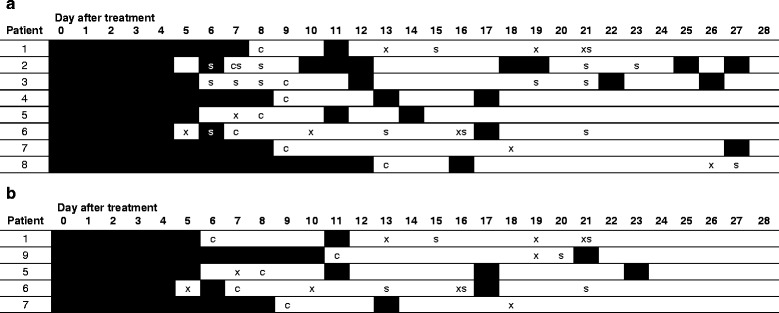
Test results, reported sexual contact and blips of *Chlamydia trachomatis* RNA (**a**) or DNA (**b**) per day of follow-up after treatment in patients with blips. Black squares: positive for *C. trachomatis* (before clearance, or blip); white squares: negative for *C. trachomatis*; c: clearance; x: missing sample; s: sexual contact reported on this day (after sampling)

When analysing all samples after clearance (411 for RNA and 403 for DNA), the median number of days at risk per patient was 19 (range 12–25) for RNA, and 20 (range 11–25) for DNA. The overall risk of finding a blip after clearance was 4.4 % per day for RNA and 1.7 % per day for DNA. DNA blips had significantly higher Ct-values (mean: 38.6, range: 35.3–41.7) compared to pre-treatment samples (mean: 31.6, range: 27.8–39.3, *P* < 0.001). Only two RNA blips and two DNA blips were observed within 24 h of reported sex, of which one was a blip in both RNA and DNA testing (Fig. 2).

Although the sample size was relatively small, we determined characteristics associated with blips using GEE logistic regression. RNA blips were significantly associated with HIV-positivity (odds ratio [OR]: 8.0, 95 % confidence interval [95 %-CI]: 2.3–28.1, *P* = 0.001), a chlamydia infection in the previous 6 months (OR: 6.6, 95 %-CI: 1.8–24.7, *P* = 0.005), and with absence of symptoms or signs (OR: 0.17, 95 %-CI: 0.03–0.97, *P* = 0.05). In multivariable analysis, HIV-status and previous chlamydia infection remained significantly associated (OR: 7.1, 95 %-CI: 2.5–19.9, *P* < 0.001, and OR: 5.9, 95 %-CI: 2.2–16.3, *P* = 0.001, respectively). As there were seven DNA blips, the power for this analysis was limited; only sexual contact in the 24 h before sampling was significantly associated in univariable analysis (OR: 6.8, 95 %-CI: 1.3–36.7, *P* = 0.03).

## Discussion

In this study we analysed the time to clearance of anogenital *C. trachomatis* after treatment in patients coinfected with *N. gonorrhoeae*, using modern RNA- and DNA-based NAATs and daily collected samples. The median time to clearance was 7 days for RNA and 6 days for DNA. Ninety-five per cent of patients had cleared *C. trachomatis* RNA and DNA after 13 and 14 days, respectively.

Several previous studies reported on in vivo clearance of *C. trachomatis* after treatment, but used different molecular testing methods, and a sampling frequency of no more than twice a week. Some studies observed clearance of DNA within 3 weeks using ligase chain reaction or in-house PCR methods [13, 14], while other studies reported 5–25 % DNA persistence after 3–4 weeks [9, 15, 16, 25]. The exact time to clearance of RNA, when tested by NAAT, was also previously unknown. Sena et al. reported 12 % RNA persistence after 4 weeks in men, while Dukers et al. reported 42 % intermittent positive results up to 51 days [9, 19]. In addition, a recent study reported 24 % positivity in 180 patients after 6 months, but no data on re-exposure or reinfections were reported [26]. Since none of these previous studies reported results from consecutive days, the exact time to clearance could not be determined, and prolonged persistence could not be distinguished from blips. In addition, none reported data on events that could have caused reinfection, like was done in the current study. Although no previous studies have been performed on the clearance of *C. trachomatis* in patients coinfected with *N. gonorrhoeae*, our results confirm the assumption that *C. trachomatis* RNA and DNA are cleared after 2 weeks [14, 17, 25].

Intermittent positive results or blips have been previously described by several studies; between 5 and 18 % of patients had a positive test result following a previous negative result after treatment [9, 14, 17–19]. We report an overall risk of blips after clearance of almost 2 % for DNA and 4 % for RNA, and no treatment failures or reinfections. The slightly higher sensitivity of the RNA test could explain the higher frequency of RNA blips, compared to DNA blips. The fact that all but one of the pre-treatment samples initially diagnosed by RNA testing were also positive for DNA, makes it unlikely that the different sensitivity is of clinical importance in diagnosing chlamydia. On the other hand, when performing a TOC, higher sensitivity could result in more positive results. The implications of this should be clarified in larger studies. Unfortunately, NAAT testing only gives information about the presence of genetic material, but not on the viability of the pathogen or whether this is still infectious. Therefore blips could be the result of deposition of viable or non-viable genetic material by a sexual partner, release of genetic material by degrading cells, or possibly the presence of elementary bodies that hold genetic components of *C. trachomatis* [18, 27]. The origin of blips needs to be further examined in future research. Due to the small sample size we could not identify characteristics associated with the occurrence of blips. However, the occurrence of blips, as well as reinfections, could explain the higher positivity rates reported by some studies, especially in those with very long follow-up and limited sampling [9, 15, 18, 19, 26].

Our study has several limitations. It was performed at a single centre in a high-incidence population, which may limit the generalizability. We selected patients from a cohort infected with *N. gonorrhoeae*, so this concerns a population coinfected with *N. gonorrhoeae* and *C. trachomatis*, which may influence both time to clearance and the occurrence of blips. Because all patients were coinfected with *N. gonorrhoeae*, they were also treated with ceftriaxone. Since ceftriaxone is not effective against chlamydia, it is unlikely that treatment with ceftriaxone influenced the clearance of *C. trachomatis*. Nevertheless, confirmation of our results in chlamydia monoinfected patients is warranted.

## Conclusions

Our results are the first to show that *C. trachomatis* RNA and DNA are cleared within 14 days of initiating treatment, using daily testing. Despite the small sample size, our results suggest that if a TOC is indicated in patients with *C. trachomatis* and *N. gonorrhoeae* coinfection, it is best performed after at least 2 weeks. Positive results obtained more than 2 weeks after initiation of treatment should be evaluated carefully, as these probably represent blips, and do not necessarily indicate treatment failure or reinfection. To exclude blips as the cause of a positive TOC, we recommend to obtain a new sample for retesting.

## Abbreviations

95 %-CI: 95 % confidence interval
﻿Ct: Cycle threshold
﻿DNA: Deoxyribonucleic acid
GEE: Generalized estimated equation
HIV: Human immunodeficiency virus
IQR: Interquartile range
MSM: Men who have sex with men
NAAT: Nucleic acid amplification test
OR: Odds ratio
PCR: Polymerase chain reaction
RNA: Ribonucleic acid
STI: Sexually transmitted infection
TOC: Test of cure
